# Interpregnancy maternal weight change is not associated with offspring weight and obesity at age 2 years

**DOI:** 10.1038/s41366-024-01554-y

**Published:** 2024-06-13

**Authors:** Kate Maslin, Lieveke Ameye, Diederik Vancoppenolle, Anne Rochtus, Hanne Van Uytsel, Jill Shawe, Roland Devlieger, Annick Bogaerts

**Affiliations:** 1 School of Nursing and Midwifery, Faculty of Health, University of Plymouth, Devon, UK; 2 REALIFE Research Group, Research Unit Woman and Child, Department of Development and Regeneration, KU Leuven, 3000 Leuven, Belgium; 3 Opgroeien Agency in the administration of the Flemish Government, Flanders, Belgium; 4 Department of Pediatrics, University Hospital Leuven, 3000 Leuven, Belgium; 5 Royal Cornwall Hospital NHS Trust, Truro, Cornwall UK; 6 Department of Obstetrics and Gynecology, University Hospital Leuven, 3000 Leuven, Belgium; 7 Department of Obstetrics and Gynecology, GZA Hospitals Sint-Augustinus, 2610 Antwerp, Belgium; 8 L-C&Y KU Leuven Child & Youth Institute, 3000 Leuven, Belgium

**Keywords:** Risk factors, Epidemiology, Paediatrics

## Abstract

**Background:**

Weight retention between pregnancies is associated with increased risk of perinatal complications, but it is unclear whether there is an association with offspring weight status. This study aimed to determine whether maternal interpregnancy weight change is associated with offspring overweight/obesity, controlling for confounding variables.

**Subjects/methods:**

Routinely collected linked data from perinatal and child datasets, in Flanders, Belgium were used. Women having their first and second live births between 2009–2018 were included. The association between maternal interpregnancy weight change and overweight/obesity in the second child at 2 years was examined by logistical regression models.

**Results:**

A total of 33,172 women were included. 52.7% (*n* = 17478) had a stable interpregnancy BMI, 24.1% (*n* = 8024) and 8.5% (*n* = 2821) had moderate and substantial BMI increases respectively. At 2 years, 91.6% (*n* = 30383) of the second offspring had a healthy weight, 0.6% (*n* = 210), 7.0% (*n* = 2312) and 0.8% (*n* = 267) were in the underweight, overweight and obesity BMI categories respectively. Multivariate analysis showed no statistical evidence that maternal interpregnancy BMI change is independently associated with overweight/obesity in the second child. The strongest independent factors were the first child (sibling) being in the obesity category at 2 years (odds ratio [OR] 7.2, [95% CI, 5.49–9.45] and being born Large for Gestational Age (LGA) (2.13 [1.92–2.37]). The following variables were also independently associated with the outcome measure: maternal African origin (1.90 [1.59–2.26]), maternal obesity at start of first pregnancy (1.33 [1.16–1.53]), excessive gestational weight gain in the second pregnancy (1.15 [1.04–1.28]), being born after a < 1-year interpregnancy time interval (1.17 [1.05–1.30]) and not being exclusively breastfed at 12 weeks old (1.29 [1.10–1.52]).

**Conclusion:**

Sibling obesity and being born LGA were most strongly independently associated with overweight/obesity at 2 years. This supports the need for family interventions and to address risk factors for development of LGA infants. There was no independent association with interpregnancy weight gain, contrary to what was hypothesised.

## Introduction

Both pregnancy and postpartum are key life stages during which a woman’s weight trajectory is altered. In order to support optimal foetal development and a healthy infant birth weight, it is recommended that those starting pregnancy in the healthy weight bracket should gain approximately 11–16 kg, with less gestational weight gain (GWG) advised for those with a higher starting body mass index (BMI) and more GWG for those with a lower starting BMI [1–4]. However, due to difficulties losing weight in the postpartum period, pregnancy itself has been suggested to contribute to the development of obesity [5, 6] with meta-analyses suggesting that multiparous women are more likely to have a higher pre-pregnancy BMI than primiparous women [7].

In Europe, the prevalence of pre-pregnancy overweight and obesity was between 30–50% and 8–26% respectively in 2014 [8]. More recent figures from the Flanders region of Belgium estimate the prevalence to be 26.4% and 15.8%, respectively [9]. It has been suggested that due to the increase in maternal obesity, GWG guidelines written in 2009 [1] may recommend *too* much weight gain in those with class II (≥35 kg/m^2^) and class III (≥40 kg/m^2^) obesity [10]. The duration and extent of postpartum weight retention is associated with pre-pregnancy weight and GWG [11–15]. Therefore, preventing excessive GWG and limiting postpartum weight retention is an important public health concern.

Postpartum weight retention may lead to interpregnancy weight increase. Research indicates that ~35% of women with a BMI in the healthy or overweight range move to a higher BMI category by the start of their next pregnancy [16]. Four systematic reviews [3, 17–19] have demonstrated that significant interpregnancy weight gain is associated with an increased risk of adverse perinatal outcomes. These include both maternal (gestational diabetes, pre-eclampsia, caesarean birth) and offspring outcomes (large for gestational age (LGA), macrosomia), but not childhood weight status. Conversely, an interpregnancy BMI decrease is associated with a decreased risk of LGA [17–19]. All four systematic reviews also demonstrated that the relative risk of interpregnancy weight gain on some adverse outcomes was greater among those with a BMI < 25 kg/m^2^ at the start of the index pregnancy compared to those with a starting BMI ≥ 25 kg/m^2^.

Although many studies have investigated the impact of pre-pregnancy maternal obesity and/or GWG on offspring weight status [20], the association between interpregnancy weight change and offspring weight status has not been explored in depth. To our knowledge, only two previous studies [21, 22] have been published, both finding a higher risk of childhood obesity in the offspring of women with interpregnancy weight gain (defined as ≥3 [22] or ≥4 [21] BMI units respectively).

We previously showed in a regional representative cohort of women from Belgium (*n* = 57897) that weight retention between the first and second pregnancy is associated with an increased risk of perinatal complications, even in those starting pregnancy with a BMI < 25 kg/m^2^ [23]. We now build on this work, using the same regional perinatal dataset [24]. We examine the effect of interpregnancy weight change on the risk of early childhood overweight and obesity, linking to an extensive early childhood dataset [25], whilst controlling for confounding variables with multivariate analysis.

## Materials and methods

### Datasets

Pregnancy and birth data is derived from the Flemish Study Center for Perinatal Epidemiology (SPE) dataset for women with 1st and 2nd parity within the study period of 2009–2018 [24]. Child data is derived from the “Opgroeien” (formerly known as “Kind & Gezin”) dataset [25]. SPE routinely registers perinatal data from all deliveries in Flanders, Belgium, containing information about maternal and gestational age at delivery, maternal height and weight before pregnancy, maternal weight at delivery, parity, diabetes in pregnancy, mode of delivery and birth weight. Opgroeien collects data about (young) children and their families in Flanders. Their database contains sociodemographic data, breastfeeding status until 6 months, and child’s height/weight measurements at birth until 2 years. Data from SPE and Opgroeien were linked by a Trusted-Third Party, matching parity, maternal and neonate date of birth, and maternity centre.

### Measurement of weight, height and interpregnancy changes

Pre-pregnancy weight (kg) and height (m) were self-reported during pregnancy. Maternal weight at delivery (kg) was measured in the delivery room or, if not available, weight at the last prenatal visit was used. BMI was calculated as weight/(height)^2^ (kg/m^2^). GWG was calculated by subtracting maternal pre-pregnancy weight from weight at delivery and was categorized as insufficient, adequate, or excessive in accordance with the Institute of Medicine guidelines [1].

Interpregnancy time interval was calculated as the number of completed weeks between the birth of the first and second neonate minus the duration of the second pregnancy (weeks) and categorised into <1, 1–1.9, 2–2.9 or >3 years. Interpregnancy weight change was calculated as the difference between the pre-pregnancy BMI of the first pregnancy and the pre-pregnancy BMI of the second pregnancy as per previous publications [23, 26]. Interpregnancy weight change category was defined as: <−1 kg/m^2^ (weight loss), −1 to 0.99 kg/m^2^ (reference/stable group), 1–2.99 kg/m^2^ (moderate increase), and ≥3 kg/m^2^ (substantial increase) [20, 22].

### Covariates

The highest maternal educational qualification was self-reported and categorised as primary education/no education, lower secondary education, secondary education, and higher education. Maternal origin was defined as maternal country of birth and recorded under fourteen categories, which were combined into three categories: Africa, Europe, and other. Living in poverty was recorded if 3 of 6 deprivation characteristics were met (income, education, employment, low stimulation, housing, and health of family).

Breastfeeding status was reported in three categories (yes exclusively, yes not exclusively, no) at three time points: 6 days; 12 weeks and 6 months. The categories were merged as follows into four categories for analysis: no breastfeeding at 6 days, exclusively breastfeeding at (least) 6 days, exclusively breastfeeding at (least) 12 weeks, and exclusively breastfeeding at 6 months.

Information about smoking during pregnancy was only available in 39% of the cases and was therefore not retained in the analyses.

### Outcome assessment

Offspring weight (g) and length (cm) were routinely measured at birth and at 2 years. Measurements took place as close to age 2 years as possible, only those whose measurements that took place between 23 and 25 months of age were included. BMI was calculated as weight/(height)^2^.

Low BMI was classified according to WHO criteria [27]. Overweight and obesity were defined using the International Obesity Task Force definition BMI *z* score cut-offs [28]. Overweight and obesity are presented as separate categories for descriptive statistics but combined as one variable for the univariate and multivariate models.

### Statistical analysis

Descriptive statistics and frequencies were calculated for all variables for all participants and stratified by interpregnancy weight change category. Unadjusted comparisons were calculated using Kruskal-Wallis for continuous variables and chi-square test for categorical variables.

To estimate the controlled direct effect of interpregnancy BMI change, potential confounders were additionally adjusted in a multivariate model. The covariates included were: maternal pre-pregnancy BMI category before first pregnancy, maternal age category at first pregnancy, maternal origin, BMI category of 1st child at 2 years, interpregnancy time interval category, GWG of 2nd pregnancy category [1], birth weight category of 2nd child, living in deprivation status and breastfeeding category of 2nd child. Maternal education was not included in the model as it was highly associated with living in deprivation status (c-index of 0.89).

The association between the maternal interpregnancy weight change and risk of childhood overweight/obesity in the second child was examined by logistic regression models predicting the outcome (overweight/obesity of the second child) by categories of BMI gain (with stable BMI as the reference category) and covariates using a logit link. This was analysed first in the whole sample and then stratified by maternal BMI category at first pregnancy. The multiple logistic regression models were complete case analyses: no data imputation for missing values in one of the explanatory variables. A statistical significance level of 0.05 with 95% confidence intervals was used in the models.

### Ethical approval

The scientific committee of SPE granted approval for the analysis of the de-identified data. This study was furthermore exempt from approval by the KU Leuven institutional review board because data is routinely collected registry data that is used for scientific purposes only. A signed protocol agreement for data sharing was signed between SPE, Opgroeien and KU Leuven to this effect on 09/07/2020. The process was facilitated by the university’s data protection office.

## Results

**Fig. 1:**
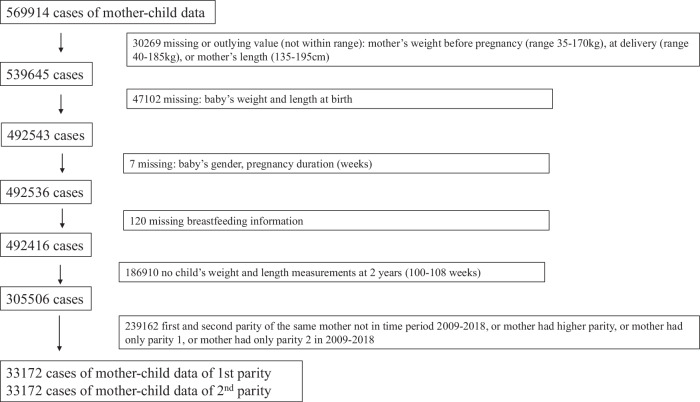
Flow chart of mother and child cases for inclusion in analysis. Pregnancy and birth data is derived from the Flemish Study Center for Perinatal Epidemiology (SPE) dataset. Child data is derived from the “Opgroeien” (formerly known as “Kind & Gezin”) dataset.

We received a dataset of 569,914 cases (mother-child data). After selecting cases with 1st and 2nd parity within the study period of 2009–2018 and after excluding cases with missing data, a total of 33,172 cases remained for analysis. The flowchart of cases is shown in Fig. 1.

### Descriptive statistics

Descriptive statistics are shown in Table 1. Mean maternal age at the second pregnancy was 30.4 (3.58) years. Most mothers were of European origin (92.5%, *n* = 30,670), most had higher education (68.3%, *n* = 21,939), with a small minority living in deprivation (4.0%, *n* = 1308). Approximately two-thirds (67.7%, *n* = 22,441) had a BMI in the healthy category at the start of pregnancy 1, decreasing to 62.8% (*n* = 20,826) at the start of pregnancy 2. A greater proportion of infants from pregnancy 2 were exclusively breastfed until 6 months, compared to the first infant (8.8% vs. 6.6%). Most offspring from pregnancy 1 were in the healthy BMI category at 2 years (92.4%, *n* = 30,656), compared to 91.6% (*n* = 30,383) for child 2.

**Table 1:** Descriptive statistics for whole dataset and stratified by interpregnancy weight change category.

	Weight decrease < −1 kg/m^2^ (*N* = 4849, 14.6%)	Stable weight −1 to 0.99 kg/m^2^ (*N* = 17,478, 52.7%)	Moderate weight increase 1 to 2.99 kg/m^2^ (*N* = 8024, 24.1%)	Substantial weight increase ≥3 kg/m^2^ (*N* = 2821, 8.5%)	Total (*N* = 33,172)	*P*-value
Maternal age, pregnancy 1						
Mean (SD)	27.8 (3.60)	28.2 (3.34)	27.7 (3.62)	26.7 (3.99)	27.9 (3.53)	<0.0001^1^
Median (IQR)	28.0 (25.0, 30.0)	28.0 (26.0, 30.0)	28.0 (25.0, 30.0)	27.0 (24.0, 29.0)	28.0 (26.0, 30.0)	
Range	14.0, 43.0	15.0, 43.0	15.0, 47.0	15.0, 45.0	14.0, 47.0	
Maternal age, pregnancy 2						<0.0001^1^
Mean (SD)	30.3 (3.67)	30.6 (3.41)	30.3 (3.68)	29.5 (4.01)	30.4 (3.58)	
Median (IQR)	30.0 (28.0, 32.0)	30.0 (28.0, 33.0)	30.0 (28.0, 33.0)	30.0 (27.0, 32.0)	30.0 (28.0, 33.0)	
Range	18.0, 47.0	17.0, 46.0	18.0, 49.0	17.0, 48.0	17.0, 49.0	
Maternal origin, *n* (%)						<0.0001^2^
Africa	187 (3.9)	412 (2.4)	456 (5.7)	275 (9.8)	1330 (4.0)	
Europe	4525 (93.4)	16,588 (94.9)	7208 (89.9)	2356 (83.7)	30,678 (92.5)	
Other	134 (2.8)	472 (2.7)	350 (4.4)	186 (6.6)	1142 (3.4)	
Maternal education, *n* (%)						<0.0001^2^
Primary education/no education	75 (1.6)	179 (1.1)	148 (1.9)	131 (4.9)	533 (1.7)	
Lower secondary education	236 (5.0)	571 (3.4)	419 (5.4)	286 (10.6)	1512 (4.7)	
Secondary education	1204 (25.7)	3636 (21.4)	2254 (29.2)	1039 (38.5)	8133 (25.3)	
Higher education	3177 (67.7)	12611 (74.2)	4909 (63.5)	1242 (46.0)	21,939 (68.3)	
Living in deprivation, child 2, *n* (%)	213 (4.4)	401 (2.3)	363 (4.5)	331 (11.8)	1308 (4.0)	<0.0001^2^
Interpregnancy time interval, *n* (%)						<0.0001^2^
<1 year	945 (19.5)	3687 (21.1)	1634 (20.4)	568 (20.1)	6834 (20.6)	
1–1.9 years	2434 (50.2)	8724 (49.9)	3521 (43.9)	1034 (36.7)	15,713 (47.4)	
2–2.9 years	1002 (20.7)	3617 (20.7)	1775 (22.1)	656 (23.3)	7050 (21.3)	
≥3 years	468 (9.7)	1450 (8.3)	1094 (13.6)	563 (20.0)	3575 (10.8)	
Maternal BMI start pregnancy 1 (kg/m^2^)						<0.0001^1^
Mean (SD)	25.4 (4.79)	22.8 (3.65)	23.8 (4.01)	25.2 (4.77)	23.6 (4.16)	
Median (IQR)	24.3 (22.1, 27.6)	22.0 (20.3, 24.3)	23.1 (21.0, 25.8)	24.5 (21.8, 27.7)	22.8 (20.8, 25.5)	
Range	16.5, 50.7	13.7, 52.6	14.7, 46.4	13.5, 50.7	13.5, 52.6	
Maternal BMI category start pregnancy 1, *n* (%)						<0.0001^2^
Underweight ( < 18.5 kg/m^2^)	52 (1.1)	960 (5.5)	350 (4.4)	118 (4.2)	1480 (4.5)	
Healthy weight (18.5–24.9 kg/m^2^)	2702 (55.7)	13,092 (74.9)	5216 (65.0)	1431 (50.7)	22,441 (67.7)	
Overweight (25.0–29.9 kg/m^2^)	1337 (27.6)	2574 (14.7)	1814 (22.6)	851 (30.2)	6576 (19.8)	
Obesity class I (30.0–34.9 kg/m^2^)	517 (10.7)	644 (3.7)	507 (6.3)	304 (10.8)	1972 (5.9)	
Obesity class II (35.0–39.9 kg/m^2^)	174 (3.6)	161 (0.9)	116 (1.4)	97 (3.4)	548 (1.7)	
Obesity class III ( ≥ 40 kg/m^2^)	67 (1.4)	47 (0.3)	21 (0.3)	20 (0.7)	155 (0.5)	
Maternal BMI start pregnancy 2 (kg/m^2^)						<0.0001^1^
Mean (SD)	23.2 (4.26)	22.8 (3.67)	25.5 (4.10)	29.7 (5.04)	24.1 (4.49)	
Median (IQR)	22.3 (20.3, 25.2)	22.1 (20.3, 24.3)	24.8 (22.7, 27.6)	29.0 (26.1, 32.6)	23.2 (21.0, 26.2)	
Range	14.5, 47.0	13.9, 52.2	16.2, 48.7	18.8, 53.9	13.9, 53.9	
Maternal BMI category start pregnancy 2, *n* (%)						<0.0001^2^
Underweight ( < 18.5 kg/m^2^)	372 (7.7)	961 (5.5)	60 (0.7)	0 (0.0)	1393 (4.2)	
Healthy weight (18.5–24.9 kg/m^2^)	3226 (66.5)	13,002 (74.4)	4121 (51.4)	477 (16.9)	20,826 (62.8)	
Overweight (25.0–29.9 kg/m^2^)	866 (17.9)	2655 (15.2)	2789 (34.8)	1136 (40.3)	7446 (22.4)	
Obesity class I (30.0–34.9 kg/m^2^)	290 (6.0)	655 (3.7)	794 (9.9)	810 (28.7)	2549 (7.7)	
Obesity class II (35.0–39.9 kg/m^2^)	78 (1.6)	153 (0.9)	217 (2.7)	276 (9.8)	724 (2.2)	
Obesity class III ( ≥ 40 kg/m^2^)	17 (0.4)	52 (0.3)	43 (0.5)	122 (4.3)	234 (0.7)	
Gestational weight gain pregnancy 1 (kg)						<0.0001^1^
Mean (SD)	11.1 (5.85)	13.1 (4.50)	14.7 (5.04)	16.7 (6.32)	13.5 (5.23)	
Median (IQR)	11.0 (8.0, 15.0)	13.0 (10.0, 16.0)	14.0 (12.0, 18.0)	16.0 (12.0, 20.0)	13.0 (10.0, 16.0)	
Range	−26.0, 37.0	−14.0, 46.0	−12.0, 49.0	−19.0, 47.0	−26.0, 49.0	
Gestational weight gain category pregnancy 1, *n* (%)						<0.0001^2^
Inadequate	1814 (37.4)	5149 (29.5)	1366 (17.0)	316 (11.2)	8645 (26.1)	
Adequate	1629 (33.6)	7656 (43.8)	3021 (37.6)	691 (24.5)	12,997 (39.2)	
Excessive	1406 (29.0)	4673 (26.7)	3637 (45.3)	1814 (64.3)	11,530 (34.8)	
Gestational weight gain pregnancy 2 (kg)						<0.0001^1^
Mean (SD)	13.4 (5.15)	12.6 (4.45)	11.8 (4.97)	9.6 (6.30)	12.3 (4.96)	
Median (IQR)	13.0 (10.0, 16.0)	12.0 (10.0, 15.0)	12.0 (9.0, 15.0)	10.0 (6.0, 14.0)	12.0 (10.0, 15.0)	
Range	−10.0, 45.0	−21.0, 47.0	−18.0, 48.0	−20.0, 39.0	−21.0, 48.0	
Gestational weight gain category pregnancy 2, *n* (%)						<0.0001^2^
Inadequate	1352 (27.9)	5822 (33.3)	2354 (29.3)	780 (27.6)	10,308 (31.1)	
Adequate	1889 (39.0)	7431 (42.5)	3037 (37.8)	926 (32.8)	13,283 (40.0)	
Excessive	1608 (33.2%)	4225 (24.2%)	2633 (32.8%)	1115 (39.5)	9581 (28.9)	
Infant 1 gestational age, *n* (%)						0.6605^2^
Term ( ≥ 37 weeks)	4600 (94.9)	16,558 (94.7)	7628 (95.1)	2683 (95.1)	31,469 (94.9)	
Preterm ( < 37 weeks)	249 (5.1)	920 (5.3)	396 (4.9)	138 (4.9)	1703 (5.1)	
Infant 2 gestational age, *n* (%)						0.7041^2^
Term ( ≥ 37 weeks)	4702 (97.0)	16,935 (96.9)	7780 (97.0)	2745 (97.3)	32,162 (97.0)	
Preterm ( < 37 weeks)	147 (3.0)	543 (3.1)	244 (3.0)	76 (2.7)	1010 (3.0)	
Sex baby 1, *n* (%)						0.4052^2^
Male	2487 (51.3%)	9011 (51.6)	4051 (50.5)	1428 (50.6)	16,977 (51.2)	
Sex baby 2, *n* (%)						0.5010^2^
Male	2439 (50.3%)	8987 (51.4)	4130 (51.5)	1432 (50.8)	16,988 (51.2)	
Birth weight category pregnancy 1, *n* (%)						0.0007^1^
Small for gestational age	394 (8.1)	1305 (7.5)	641 (8.0)	231 (8.2)	2571 (7.8)	
Appropriate for gestational age	3894 (80.3)	14,298 (81.8)	6408 (79.9)	2232 (79.1)	26,832 (80.9)	
Large for gestational age	561 (11.6)	1875 (10.7)	975 (12.2)	358 (12.7)	3769 (11.4)	
Birth weight category pregnancy 2, *n* (%)						<0.0001^1^
Small for gestational age	401 (8.3)	1319 (7.5)	582 (7.3)	180 (6.4)	2482 (7.5)	
Appropriate for gestational age	3932 (81.1)	14,260 (81.6)	6425 (80.1)	2241 (79.4)	26,858 (81.0)	
Large for gestational age	516 (10.6)	1899 (10.9)	1017 (12.7)	400 (14.2)	3832 (11.6)	
Feeding infant 1, *n* (%)						<0.0001^2^
Exclusively BF at 6 months	339 (7.0)	1170 (6.7)	498 (6.2)	177 (6.3)	2184 (6.6)	
Exclusively BF at (least) 12 weeks	1273 (26.3)	5106 (29.3)	2016 (25.1)	666 (23.6)	9061 (27.3)	
Exclusively BF at (least) 6 days	1827 (37.8)	6678 (38.3)	3128 (39.0)	1018 (36.1)	12,651 (38.2)	
Not exclusively BF at 6 days	1400 (28.9)	4502 (25.8)	2376 (29.6)	959 (34.0)	9237 (27.9)	
Feeding infant 2, *n* (%)						<0.0001^2^
Exclusively BF at 6 months	435 (9.0)	1561 (8.9)	675 (8.4)	250 (8.9)	2921 (8.8)	
Exclusively BF at (least) 12 weeks	1159 (23.9)	4621 (26.5)	1848 (23.1)	590 (21.0)	8218 (24.8)	
Exclusively BF at (least) 6 days	1575 (32.5)	5795 (33.2)	2608 (32.5)	878 (31.2)	10,856 (32.8)	
Not exclusively BF at 6 days	1675 (34.6)	5469 (31.3)	2882 (36.0)	1098 (39.0)	11,124 (33.6)	
BMI child 1 at 2 years (kg/m^2^)						<0.0001^1^
Mean (SD)	16.2 (1.29)	16.2 (1.29)	16.2 (1.40)	16.3 (1.41)	16.2 (1.33)	
Median (IQR)	16.2 (15.4, 17.0)	16.1 (15.3, 17.0)	16.1 (15.3, 17.0)	16.2 (15.4, 17.2)	16.1 (15.3, 17.0)	
Range	11.9, 22.0	11.3, 39.0	10.9, 54.9	12.3, 22.7	10.9, 54.9	
BMI category child 1 at 2 years, *n* (%)						<0.0001^2^
Underweight BMI	40 (0.8)	158 (0.9)	76 (0.9)	25 (0.9)	299 (0.9)	
Healthy BMI	4497 (92.7)	16,301 (93.3)	7334 (91.4)	2524 (89.5)	30,656 (92.4)	
Overweight BMI	285 (5.9)	907 (5.2)	547 (6.8)	234 (8.3)	1973 (5.9)	
Obese BMI	27 (0.6)	112 (0.6)	67 (0.8)	38 (1.3)	244 (0.7)	
BMI child 2 at 2 years (kg/m^2^)						<0.0001^1^
Mean (SD)	16.4 (1.50)	16.3 (1.31)	16.3 (1.34)	16.5 (1.40)	16.3 (1.35)	
Median (IQR)	16.3 (15.5, 17.2)	16.2 (15.4, 17.1)	16.2 (15.4, 17.2)	16.4 (15.5, 17.3)	16.2 (15.4, 17.1)	
Range	11.9, 53.3	12.0, 52.9	12.1, 36.3	12.2, 23.8	11.9, 53.3	
BMI category child 2 at 2 years, *n* (%)						<0.0001^2^
Underweight BMI	27 (0.6)	117 (0.7)	45 (0.6)	21 (0.7)	210 (0.6)	
Healthy BMI	4408 (90.9)	16,135 (92.3)	7332 (91.4)	2508 (88.9)	30,383 (91.6)	
Overweight BMI	370 (7.6)	1120 (6.4)	577 (7.2)	245 (8.7)	2312 (7.0)	
Obese BMI	44 (0.9)	106 (0.6)	70 (0.9)	47 (1.7)	267 (0.8)	

^1^Kruskal-Wallis *p*-value; ^2^Chi-Square *p*-value.

*BF* breastfeeding, *BMI* body mass index.

Of the 33,172 women in the dataset, 52.7% (*n* = 17,478) had a stable interpregnancy weight, whilst 24.1% (*n* = 8024) and 8.5% (*n* = 2821) had moderate and substantial increases respectively. 14.6% (*n* = 4849) experienced >1 kg/m^2^ decrease in BMI between pregnancies. Those who had substantial interpregnancy BMI increase, were more likely to be younger, of non-European background, of lower educational level and have a higher starting BMI at pregnancy 1 (*p* < 0.0001).

**Fig. 2:**
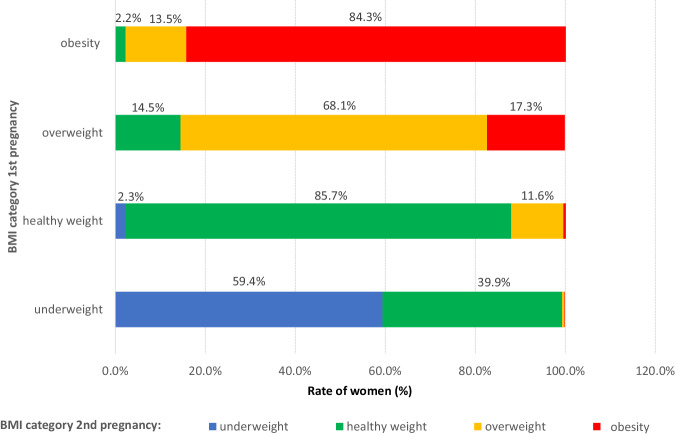
Change in maternal pre pregnancy body mass index category from first to second pregnancy. Figure shows change in pre pregnancy BMI category e.g., 11.6% of women with a healthy pre pregnancy BMI at 1st parity shift to the overweight category by the second pregnancy.

Figure 2 shows the change in BMI categories from first to second pregnancy (*n* = 33,172). It can be seen that 11.6% of women with a healthy pre pregnancy BMI at 1st parity move to the overweight category by the second pregnancy, 17.3% of those in the overweight category move to the obesity category.

Univariate analysis exploring association to weight status of the second child at age 2 years is shown in Supplementary file 1.

### Multivariate analysis

Table 2 shows the multivariate models investigating variables associated with overweight/obesity in the 2nd child at 2 years, across the whole sample and stratified by maternal BMI category at the start of the first pregnancy. There was no statistical evidence that maternal interpregnancy BMI change is independently associated with overweight/obesity in the 2nd child at 2 years.

**Table 2:** Associations of interpregnancy BMI change with risk of childhood overweight/ obesity, stratified by BMI category in the first pregnancy.

			Maternal BMI category at start 1st pregnancy					
	Full sample (*N* = 33,172)		Healthy BMI category (*N* = 22,441)		Overweight BMI category (*N* = 6576)		Obese BMI category (*N* = 2675)	
	OR	95% CI	OR	95% CI	OR	95% CI	OR	95% CI
Interpregnancy BMI change category								
<−1 kg/m^2^ (weight loss)	1.11	0.98 to 1.25	1.10	0.93 to 1.30	1.04	0.82 to 1.30	1.14	0.82 to 1.57
−1 to 0.99 kg/m^2^ unit (stable)	Ref		Ref		Ref		Ref	
1 to 2.99 kg/m^2^ (moderate increase)	1.01	0.91 to 1.12	1.08	0.95 to 1.23	0.87	0.70 to 1.07	0.98	0.70 to 1.37
≥3 kg/m^2^ (substantial increase)	1.13	0.98 to 1.31	1.18	0.96 to 1.45	0.96	0.73 to 1.26	1.16	0.80 to 1.69
Maternal BMI category start pregnancy 1								
Underweight ( < 18.5 kg/m^2^)	**0.60**	**0.45 to 0.79**						
Healthy weight (18.5–24.9 kg/m^2^)	Ref							
Overweight (25.0–29.9 kg/m^2^)	**1.19**	**1.07 to 1.32**						
Obesity ( ≥ 30.0 kg/m^2^)	**1.33**	**1.16 to 1.53**						
Maternal age pregnancy 2 (years)								
<25	1.13	0.94 to 1.37	0.98	0.76 to 1.28	**1.73**	**1.21 to 2.47**	0.92	0.54 to 1.57
25–29	Ref		Ref		Ref		Ref	
30–34	0.94	0.86 to 1.03	0.95	0.84 to 1.07	1.01	0.84 to 1.22	0.80	0.60 to 1.05
≥35	1.03	0.90 to 1.19	0.98	0.82 to 1.17	1.13	0.86 to 1.49	1.01	0.69 to 1.46
Maternal origin								
Europe	Ref		Ref		Ref		Ref	
Africa	**1.90**	**1.59 to 2.26**	**1.85**	**1.46 to 2.33**	**2.53**	**1.83 to 3.51**	1.58	0.95 to 2.64
Other	**1.27**	**1.02 to 1.58**	1.25	0.96 to 1.63	**1.68**	**1.07 to 2.64**	1.16	0.50 to 2.70
BMI category child 1 at 2 years								
Underweight	**0.06**	**0.01 to 0.41**	**0.08**	**0.01 to 0.58**	*	*	*	*
Healthy	Ref		Ref		Ref		Ref	
Overweight	**3.89**	**3.46 to 4.37**	**4.18**	**3.59 to 4.87**	**3.29**	**2.61 to 4.14**	**4.17**	**3.05 to 5.69**
Obesity	**7.20**	**5.49 to 9.45**	**9.50**	**6.51 to 13.88**	**6.90**	**4.22 to 11.28**	**4.28**	**2.14 to 8.58**
Interpregnancy time interval								
<1 year	**1.17**	**1.05 to 1.30**	**1.26**	**1.11 to 1.45**	**1.07**	**0.86 to 1.33**	0.98	0.71 to 1.35
1–1.9 years	Ref		Ref		Ref		Ref	
2–2.9 years	0.93	0.83 to 1.04	1.01	0.88 to 1.17	0.79	0.63 to 1.01	0.85	0.61 to 1.17
≥3 years	1.08	0.94 to 1.24	1.09	0.91 to 1.31	**1.32**	**1.01 to 1.73**	0.81	0.54 to 1.22
Gestational Weight Gain category pregnancy 2								
Inadequate	0.92	0.83 to 1.03	0.89	0.79 to 1.01	1.06	0.81 to 1.39	0.94	0.65 to 1.36
Adequate	Ref		Ref		Ref		Ref	
Excessive	**1.15**	**1.04 to 1.28**	**1.16**	**1.01 to 1.32**	1.19	0.98 to 1.44	1.06	0.80 to 1.41
Birth weight category infant 2								
Small for gestational age	**0.45**	**0.35 to 0.57**	**0.45**	**0.34 to 0.60**	**0.38**	**0.22 to 0.67**	**0.42**	**0.18 to 0.97**
Appropriate for gestational age	Ref		Ref		Ref		Ref	
Large for gestational age	**2.13**	**1.92 to 2.37**	**2.04**	**1.77 to 2.34**	**2.35**	**1.94 to 2.86**	**2.14**	**1.62 to 2.81**
Child 2 living in deprivation								
No	Ref		Ref		Ref		Ref	
Yes	1.02	0.83 to 1.25	1.09	0.82 to 1.43	0.78	0.52 to 1.15	1.04	0.62 to 1.75
Feeding category infant 2								
Exclusively BF at 6 months	Ref		Ref		Ref		Ref	
Exclusively BF at 12 weeks (but not anymore at 6 months)	1.14	0.96 to 1.35	1.04	0.85 to 1.28	1.29	0.88 to 1.87	1.80	0.94 to 3.45
Exclusively BF at 6 days (but not anymore at 12 weeks)	**1.29**	**1.10 to 1.52**	**1.24**	**1.02 to 1.51**	**1.61**	**1.13 to 2.31**	1.36	0.72 to 2.56
No (exclusively) BF at 6 days	**1.19**	**1.01 to 1.41**	1.01	0.82 to 1.23	**1.66**	**1.17 to 2.37**	1.79	0.97 to 2.33

Significant association is shown in bold text.

Logistic regression modeling was a complete case analysis: 33,027 of the 33,172 cases (99.6%).

*BMI* body mass index, *BF* breastfeeding.

*Not possible to estimate as none of the 1st children with underweight at 24 months had a sibling with overweight or obesity at 24 months.

The most important factors in the multivariate model were the BMI of the 1st child at 2 years and the birth weight category of the 2nd child. If the first child was overweight at two years, the odds ratio (OR) of the second child being in the overweight/obesity category was 3.89 (95% CI 3.46–4.37, *p* < 0.001), increasing to 7.20 (95% CI 5.49−9.45, *p* < 0.001) if the first child was in the obesity category. This effect was strongest in the offspring of mothers who had a healthy BMI at the start of the first pregnancy (OR 9.50, 95% CI 6.51–13.88, *p* < 0.001), however, the confidence intervals are quite wide. We noted that the overweight/obesity rate of 2nd children is much lower (1555/22441, 7%) if the mother had a healthy BMI at the start of the first pregnancy, compared to if the mother had a BMI in the obesity category at the start of the first pregnancy (317/2675, 12%) (data not shown). Infants who were born LGA were 2.13 times more likely to be in the overweight/obesity BMI category at 2 years, compared to those born in appropriate gestational weight category (95% CI 1.92–2.37, *p* < 0.001).

Maternal BMI at the start of the first pregnancy was significantly associated with childhood overweight/obesity of the 2nd child at 2 years across all BMI categories. Those in the underweight category were 40% less likely to have the second child in the overweight/obesity category at 2 years (OR 0.60, 95% CI 0.45–0.79, *p* < 0.001). The OR incrementally increased with increasing maternal BMI category (OR 1.19, 95% CI 1.07–1.32, *p* = 0.001 for overweight and OR 1.33 (95% CI 1.16–1.53, *p* < 0.001) for obesity category respectively.

Children of non-European origin were more likely to be in the overweight/obesity category at age of two years, compared to those of European origin (OR of 1.90 95% CI 1.59–2.26, *p* < 0.001 for African origin and OR of 1.27 (95% CI 1.02–1.58, *p* = 0.03) for those of other origin). Those who had excessive GWG in the second pregnancy (OR 1.15, 95% CI 1.04–1.28, *p* = 0.005) were more likely to have a second child in the overweight/obesity category at 2 years. Offspring born after a short (<1 year) interpregnancy time interval (OR 1.17, 95% CI 1.05–1.30, *p* = 0.004) were more likely to be in the overweight/obesity category at age 2 years, overall and across all maternal BMI categories.

Children who were not exclusively breastfed at 6 days or exclusively breastfed for <12 weeks, were more likely to be in the overweight/obesity category at 2 years compared to those who were exclusively breastfed at 6 months (OR 1.19, 95% CI 1.01–1.41, and 1.29, 95% CI 1.10–1.52, *p* = 0.04).

## Discussion

The aim of this study was to explore patterns of interpregnancy weight change and their association with offspring weight status at age 2 years, using routinely collected data from pregnant women in Belgium. In contrast to previous findings [21, 22] and hypothesis that substantial interpregnancy weight gives a higher risk for obesity of the offspring, we did not observe this in the multivariate analysis. There was no statistical evidence that maternal interpregnancy BMI change is independently associated with overweight/obesity of the second child at 2 years. The most important factors were the BMI of the first child (sibling) at 2 years and the 2nd child being born LGA of the 2nd child. An interpregnancy interval <1 year, short or no breastfeeding, maternal pre-pregnancy BMI, excessive GWG during the second pregnancy and maternal origin have lesser, but significant independent effects on the likelihood of overweight/obesity at age two years.

Although there have been several systematic reviews conducted on the effect of interpregnancy weight change on perinatal outcomes, there is limited research on the effect on childhood weight outcomes. Only two studies have been identified [21, 22], both found increased likelihood of childhood overweight/obesity associated with substantial interpregnancy weight gain. Ziauddeen et al. [22] noted a 28.3% rate of overweight/obesity in offspring born to mothers with substantial interpregnancy weight gain, compared to 19.1% if born to a mother with stable interpregnancy weight. The study, based in the South of England (*n* = 4789) [22], assessed weight at 4–5 years, which likely explains the higher rates of overweight/obesity observed compared to the current study. Additionally, the rates of substantial interpregnancy weight change between the two studies differed considerably (23.7% compared to only 8.5% of the population in the present analysis) and the present analysis also included women who were underweight and those who had lost weight between pregnancies [22]. The multivariate model used different covariates to the present study, not accounting for GWG and breastfeeding status after 6 weeks. Similarly, the study by Adane et al. [21] (*n* = 714), based in Australia, assessed childhood weight at age 6–7 years and did not consider the effect of breastfeeding or GWG either. The potential for maternal factors, including interpregnancy weight change to have an independent influential effect on weight status in *later* childhood in this cohort, cannot be ruled out, given that the outcome measure was assessed at a relatively young stage in early childhood (2 years) and the rate of childhood overweight & obesity at age 2 years (7.8%) is likely to increase into older childhood. Indeed, rates of overweight/obesity in 6-year-old boys and girls in this region were 11.0% and 15.7% respectively in 2015, increasing to 15.5% and 18.4% at age 10 years respectively [29]. This trend has been observed in research analysing BMI trajectories from 2 to 6 years, showing that children with a high and increasing BMI trajectory between these ages are strongly associated with maternal prepregnancy obesity and overweight [30].

The finding that the BMI of the first child was the strongest predictor (OR 7.2) of the second child having a BMI in the overweight/obesity category indicates the strength of influence of shared familial factors and emphasises the clustering of overweight/obesity within families. Sibling weight status is influential due to shared common factors, namely genetics, intrauterine environment, socioeconomic status, nutrition intake and physical activity behaviour [31]. A study using a national sample of American households (*n* = 10,244) reported an increased OR of 2.2 of childhood obesity in one child households if a parent was living with obesity. However, the OR increased to 5.5 in those who had an elder sibling with obesity [32].

Being born LGA was the second most influential factor on likelihood of overweight/obesity at 2 years. This was more likely in those who had substantial interpregnancy weight gain (14.2%) compared to those who had a stable interpregnancy weight (10.9%), also shown in our previous study [23]. Longitudinal birth registry data from Canada (*n* = 81,226) found that when predicting overweight/obesity at 4–6 years, the adjusted attributable risk for LGA was 39.4% [33]. Indeed, a systematic review that examined risk factors for childhood obesity in the first 1000 days concluded that regardless of exposure definition, higher birth weight and later childhood overweight were consistently linked [34]. Certainly, pre-pregnancy weight, GWG, interpregnancy weight gain, LGA and childhood obesity all appear to be inextricably linked [2, 3, 17–19, 34, 35]. Cnattingius et al. [36] labelled the transmission of a mother’s birth weight to maternal obesity and a subsequent LGA infant a “vicious circle across generations”, calling for a focus on prevention of LGA births to curtail the intergenerational cycle of obesity. This is reflected in the WHO report “Ending Childhood Obesity”, which has embedded preconception and pregnancy care as one of six key areas for action [37].

The analysis showed that a short (<1 year) interpregnancy time interval is *independently* associated with childhood weight status at age 2 in the multivariate model, which to our knowledge, is a novel finding. A recent systematic review of >46 million pregnancies determined that an interpregnancy interval of between 18 and 23 months may be associated with potential benefits for both mothers and infants, with shorter and longer intervals being associated with adverse perinatal outcomes [38]. A separate systematic review [39] reported that longer but not shorter interpregnancy intervals were positively associated with adiposity in second or higher order children [39]. Investigating risk related to interpregnancy time interval can be difficult due to confounding factors (e.g., age, socioeconomic status, or reproductive history) [40]. The potential mechanism by which a short interpregnancy interval may be independently associated with childhood overweight/obesity is unclear and requires further investigation. This may be related to retained postpartum weight from the first pregnancy/GWG, or breastfeeding, however these aspects were controlled for in the multivariate model. Additionally, it could be related to unknown socioeconomic or environmental factors associated with having two young offspring very close in age that potentially effects dietary intake, physical activity, or sleep patterns, however, this is speculative.

The finding that a shorter duration of exclusive breastfeeding was independently associated with increased likelihood of childhood overweight or obesity, compared to 6 months exclusive breastfeeding supports the knowledge that breastfeeding is protective against childhood overweight and obesity [41], even when controlling for confounding by socioeconomic status [41]. Overall, 8.8% (*n* = 2921) of second infants were breastfed exclusively at 6 months, compared to 6.6% (*n* = 2184) of first infants. A systematic review, which focused specifically on breastfeeding and the risk of overweight with an emphasis on sibling pair studies [42] found moderate evidence that ever, compared with never, consuming human milk is associated with a lower risk of overweight and obesity at ≥2 years, particularly if the duration of human milk consumption is >6 months. Due to the number of variables when collecting data on breastfeeding, it was necessary to condense information into four categories for our analysis. This may limit the applicability of the findings as the categories are defined by exclusive breastfeeding at arbitrary timepoints. In addition, the dataset did not include information on timing of introduction of solid food, which affects the definition of exclusive breastfeeding.

Children of non-European origin were more likely to be in the overweight/obesity category at the age of two years, compared to those of European origin. This trend has previously been reported in other countries [43, 44]. We have not explored the ethnic origin differences in detail, as the aim of this manuscript was to focus moreso on potential *modifiable* factors associated with childhood weight status. Although many socioeconomic factors were controlled for, the observed ethnic differences may be affected by unmeasured residual confounding. Potentially those of African and other non-European ethnicity may have had higher rates of refugee status, with downstream effects on childhood obesity due to food insecurity, difficulty accessing healthcare, and intergenerational trauma [45].

### Strengths and limitations

The strengths of this research are the use of a large, well-characterised dataset from routinely collected data, ensuring wide population coverage that is representative of the Flanders region. Standardised objective data collection methods were used, plus the use of international standardised weight status cut-offs [27, 28]. The analysis considered multiple potentially confounding variables. Unfortunately, a small amount of relevant data was incomplete or unavailable (i.e., smoking and diabetes in pregnancy). Preconception weight was self-reported as this is not routinely monitored in women of reproductive age who may be considering pregnancy. Although measured weight is preferable, self-report weight is a practical approach, with a small magnitude of error [46]. A key limitation was the lack of information on paternal characteristics and any intergenerational or epigenetic effects.

### Clinical and research implications

The relatively short time interval selected for the measurement of weight status (2 years) in our analysis and the resulting relatively low prevalence of overweight/obesity (8.4%) may imply that the full effect of early life influences on overweight/obesity risk had not yet manifested, however, it remains critically important to investigate early modifiable risk factors for childhood obesity so that they can be better and more timely managed in a clinical setting [37]. Overall, these findings highlight the need to address risk factors for development of LGA infants, encouragement of continued exclusive breastfeeding, and for a familial approach to tackle childhood obesity [37, 47, 48]. Further research on the link between short interpregnancy intervals and childhood weight status is also warranted.

## Conclusion

In conclusion, this study using routinely collected pregnancy and early childhood data from a large sample in Belgium found no statistical evidence that maternal interpregnancy BMI change is independently associated with overweight/obesity of the second child at 2 years. In the multivariate analysis, the most important factors were the BMI of the first child (sibling) at 2 years and being born LGA. An interpregnancy time interval of <1-year, shorter breastfeeding periods, maternal origin, maternal BMI pre pregnancy and excessive GWG during the second pregnancy also had a lesser, but significant independent effect on the likelihood of overweight/obesity at two years. Despite the lack of association observed between interpregnancy weight change and childhood weight status at age 2 years, the evidence from a wealth of literature has indicated the adverse effects of substantial interpregnancy weight increase on *perinatal* outcomes [3, 17–19]. The interpregnancy time frame therefore remains a critical window of opportunity for focusing on preconception health behaviours for the next pregnancy, both for the mother and the whole family.

## Supplementary information

Supplementary file 1 univariate analysis

## Data Availability

The data that support the findings of this study are available from the Flemish Study Center for Perinatal Epidemiology (SPE) and “Opgroeien”, (formerly known as “Kind & Gezin”), but restrictions apply to the availability of these data, which were used under license for the current study, and so are not publicly available. Data are however available from the authors upon reasonable request and with permission of SPE and Opgroeien.
