# Supplementary material for: Interpregnancy maternal weight change is not associated with offspring weight and obesity at age 2 years
Source: Int J Obes (Lond). 2024 Jun 13;48(10):1402–13. doi: 10.1038/s41366-024-01554-y (PMC11420072; doi:10.1038/s41366-024-01554-y)
Supplement: Supplementary file 1 — univariate analysis [file 41366_2024_1554_MOESM1_ESM.rtf]

	
	BMI classification of second child at 2 years			
	Underweight BMI
(N=210)	Healthy BMI
(N=30383)	Overweight BMI
(N=2312)	Obese BMI
(N=267)	Total
(N=33172)	P-value	
Maternal age, pregnancy 1						0.00641	
Mean (SD)	27.6 (4.03)	27.9 (3.51)	27.7 (3.69)	27.5 (3.88)	27.9 (3.53)		
Median (IQR)	27.0 (25.0, 30.0)	28.0 (26.0, 30.0)	28.0 (25.0, 30.0)	27.0 (25.0, 30.0)	28.0 (26.0, 30.0)		
Range	18.0, 43.0	14.0, 47.0	16.0, 41.0	18.0, 38.0	14.0, 47.0		
Maternal age, pregnancy 2						0.01081	
Mean (SD)	30.1 (4.09)	30.4 (3.56)	30.2 (3.73)	30.1 (3.85)	30.4 (3.58)		
Median (IQR)	30.0 (28.0, 33.0)	30.0 (28.0, 33.0)	30.0 (28.0, 32.0)	30.0 (27.0, 33.0)	30.0 (28.0, 33.0)		
Range	20.0, 46.0	17.0, 49.0	19.0, 43.0	19.0, 40.0	17.0, 49.0		
Maternal age, pregnancy 1 (years), n (%)						<.00012	
<25	40 (19.0)	4553 (15.0)	431 (18.6)	62 (23.2)	5086 (15.3)		
25-29	104 (49.5)	16847 (55.4)	1219 (52.7)	123 (46.1)	18293 (55.1)		
30-34	58 (27.6)	7814 (25.7)	572 (24.7)	73 (27.3)	8517 (25.7)		
 ≥35	8 (3.8)	1169 (3.8)	90 (3.9)	9 (3.4)	1276 (3.8)		
Maternal age, pregnancy 2 (years), n (%)						0.00022	
<25	16 (7.6)	1353 (4.5)	138 (6.0)	18 (6.7)	1525 (4.6)		
25-29	75 (35.7)	10857 (35.7)	867 (37.5)	102 (38.2)	11901 (35.9)		
30-34	92 (43.8)	14600 (48.1)	1023 (44.2)	109 (40.8)	15824 (47.7)		
≥35	27 (12.9)	3573 (11.8)	284 (12.3)	38 (14.2)	3922 (11.8)		
Maternal origin, n (%)						<.00012	
Africa	5 (2.4)	1112 (3.7)	175 (7.6)	38 (14.3)	1330 (4.0)		
Europe	182 (86.7)	28240 (93.0)	2041 (88.4)	215 (80.8)	30678 (92.5)		
Other	23 (11.0)	1013 (3.3)	93 (4.0)	13 (4.9)	1142 (3.4)		
Maternal education, n (%)						<.00012	
Primary education/no education	5 (2.5)	455 (1.5)	59 (2.6)	14 (5.6)	533 (1.7)		
Lower secondary education	19 (9.6)	1325 (4.5)	146 (6.5)	22 (8.8)	1512 (4.7)		
Secondary education	49 (24.7)	7393 (25.1)	615 (27.6)	76 (30.3)	8133 (25.3)		
Higher education	125 (63.1)	20264 (68.8)	1411 (63.2)	139 (55.4)	21939 (68.3)		
Living in deprivation, child 2, n (%)	14 (6.7)	1140 (3.8)	127 (5.5)	27 (10.1)	1308 (4.0)	<.00012	
Interpregnancy time interval, n (%)						0.00032	
<1 year	46 (21.9)	6175 (20.3)	546 (23.6)	67 (25.1)	6834 (20.6)		
1- 1.9 years	93 (44.3)	14445 (47.5)	1070 (46.3)	105 (39.3)	15713 (47.4)		
2 -2.9 years	43 (20.5)	6513 (21.4)	439 (19.0)	55 (20.6)	7050 (21.3)		
≥3 years 	28 (13.3)	3250 (10.7)	257 (11.1)	40 (15.0)	3575 (10.8)		
Maternal BMI start pregnancy 1 (kg/m2)						<.00011	
Mean (SD)	22.4 (3.81)	23.5 (4.12)	24.6 (4.42)	25.6 (4.56)	23.6 (4.16)		
Median (IQR)	21.7 (19.8, 23.8)	22.7 (20.7, 25.3)	23.7 (21.5, 26.7)	24.6 (22.3, 27.6)	22.8 (20.8, 25.5)		
Range	15.4, 38.5	13.5, 52.6	14.7, 46.4	16.8, 41.1	13.5, 52.6		
Maternal BMI category start pregnancy 1, n (%)						<.00012	
Underweight (<18.5 kg/m2)	23 (11.0)	1401 (4.6)	53 (2.3)	3 (1.1)	1480 (4.5)		
Healthy weight (18.5–24.9 kg/m2)	148 (70.5)	20738 (68.3)	1413 (61.1)	142 (53.2)	22441 (67.7)		
Overweight (25.0–29.9 kg/m2)	27 (12.9)	5898 (19.4)	569 (24.6)	82 (30.7)	6576 (19.8)		
Obesity class I (30.0-34.9 kg/m2)	10 (4.8)	1724 (5.7)	209 (9.0)	29 (10.9)	1972 (5.9)		
Obesity class II (35.0-39.9 kg/m2)	2 (1.0)	490 (1.6)	49 (2.1)	7 (2.6)	548 (1.7)		
Obesity class III (≥40 kg/m2)	0 (0.0)	132 (0.4)	19 (0.8)	4 (1.5)	155 (0.5)		
Maternal BMI start pregnancy 2 (kg/m2)						<.00011	
Mean (SD)	22.9 (4.28)	24.0 (4.45)	25.2 (4.79)	26.6 (4.87)	24.1 (4.49)		
Median (IQR)	22.3 (19.9, 24.8)	23.1 (20.9, 26.1)	24.2 (21.8, 27.5)	25.5 (22.9, 29.4)	23.2 (21.0, 26.2)		
Range	15.1, 41.4	13.9, 53.9	15.2, 52.7	18.6, 45.3	13.9, 53.9		
Maternal BMI category start pregnancy 2, n (%)						<.00012	
Underweight (<18.5 kg/m2)	17 (8.1)	1320 (4.3)	56 (2.4)	0 (0.0)	1393 (4.2)		
Healthy weight (18.5–24.9 kg/m2)	145 (69.0)	19290 (63.5)	1269 (54.9)	122 (45.7)	20826 (62.8)		
Overweight (25.0–29.9 kg/m2)	30 (14.3)	6695 (22.0)	637 (27.6)	84 (31.5)	7446 (22.4)		
Obesity class I (30.0-34.9 kg/m2)	15 (7.1)	2232 (7.3)	259 (11.2)	43 (16.1)	2549 (7.7)		
Obesity class II (35.0-39.9 kg/m2)	1 (0.5)	641 (2.1)	67 (2.9)	15 (5.6)	724 (2.2)		
Obesity class III (≥40 kg/m2)	2 (1.0)	205 (0.7)	24 (1.0)	3 (1.1)	234 (0.7)		
Interpregnancy BMI change (kg/m2)						0.00071	
Mean (SD)	0.6 (1.92)	0.5 (1.93)	0.6 (2.12)	1.0 (2.43)	0.5 (1.95)		
Median (IQR)	0.3 (-0.4, 1.5)	0.3 (-0.4, 1.4)	0.4 (-0.4, 1.5)	0.7 (-0.3, 2.4)	0.3 (-0.4, 1.4)		
Range	-9.4, 8.0	-31.4, 17.0	-13.2, 12.1	-10.0, 9.4	-31.4, 17.0		
Interpregnancy BMI change category, n (%)						<.00012	
<-1 BMI unit	27 (12.9)	4408 (14.5)	370 (16.0)	44 (16.5)	4849 (14.6)		
-1 to 0.99 BMI unit	117 (55.7)	16135 (53.1)	1120 (48.4)	106 (39.7)	17478 (52.7)		
1 to 2.99 BMI unit	45 (21.4)	7332 (24.1)	577 (25.0)	70 (26.2)	8024 (24.2)		
≥ 3 BMI units	21 (10.0)	2508 (8.3)	245 (10.6)	47 (17.6)	2821 (8.5)		
Gestational weight gain pregnancy 1 (kg)						<.00011	
Mean (SD)	12.5 (5.38)	13.4 (5.21)	14.0 (5.40)	14.3 (5.72)	13.5 (5.23)		
Median (IQR)	12.0 (10.0, 15.0)	13.0 (10.0, 16.0)	14.0 (11.0, 17.0)	14.0 (11.0, 17.0)	13.0 (10.0, 16.0)		
Range	-3.0, 49.0	-26.0, 47.0	-19.0, 40.0	-3.0, 36.0	-26.0, 49.0		
Gestational weight gain pregnancy 1 category, n (%)						<.00012	
Inadequate	77 (36.7)	8049 (26.5)	474 (20.5)	45 (16.9)	8645 (26.1)		
Adequate	86 (41.0)	11973 (39.4)	840 (36.3)	98 (36.7)	12997 (39.2)		
Excessive	47 (22.4)	10361 (34.1)	998 (43.2)	124 (46.4)	11530 (34.8)		
Gestational weight gain pregnancy 2 (kg)						<.00011	
Mean (SD)	11.1 (4.61)	12.2 (4.93)	12.7 (5.20)	12.5 (6.06)	12.3 (4.96)		
Median (IQR)	11.0 (9.0, 14.0)	12.0 (10.0, 15.0)	13.0 (10.0, 16.0)	12.0 (9.0, 16.0)	12.0 (10.0, 15.0)		
Range	-9.0, 24.0	-21.0, 48.0	-10.0, 45.0	-8.0, 39.0	-21.0, 48.0		
Gestational weight gain category pregnancy 2, n (%)						<.00012	
Inadequate	92 (43.8)	9572 (31.5)	584 (25.3)	60 (22.5)	10308 (31.1)		
Adequate	82 (39.0)	12223 (40.2)	873 (37.8)	105 (39.3)	13283 (40.0)		
Excessive	36 (17.1)	8588 (28.3)	855 (37.0)	102 (38.2)	9581 (28.9)		
Infant 1 gestational age (weeks)						0.00021	
Mean (SD)	38.9 (1.53)	39.1 (1.48)	39.2 (1.41)	39.2 (1.64)	39.1 (1.48)		
Median (IQR)	39.0 (38.0, 40.0)	39.0 (38.0, 40.0)	39.0 (39.0, 40.0)	40.0 (38.0, 40.0)	39.0 (38.0, 40.0)		
Range	32.0, 41.0	24.0, 42.0	29.0, 42.0	30.0, 42.0	24.0, 42.0		
Infant 1 gestational age, category, n (%)						0.82532	
Term (≥37 weeks)	198 (94.3)	28815 (94.8)	2202 (95.2)	254 (95.1)	31469 (94.9)		
Preterm (<37 weeks)	12 (5.7)	1568 (5.2)	110 (4.8)	13 (4.9)	1703 (5.1)		
Infant 2 gestational age (weeks)						0.58321	
Mean (SD)	38.9 (1.40)	39.1 (1.26)	39.1 (1.29)	39.1 (1.22)	39.1 (1.26)		
Median (IQR)	39.0 (38.0, 40.0)	39.0 (38.0, 40.0)	39.0 (38.0, 40.0)	39.0 (38.0, 40.0)	39.0 (38.0, 40.0)		
Range	31.0, 41.0	26.0, 42.0	25.0, 41.0	33.0, 41.0	25.0, 42.0		
Infant 2 gestational age, category, n (%)						0.26382	
Term (≥37 weeks)	200 (95.2)	29452 (96.9)	2248 (97.2)	262 (98.1)	32162 (97.0)		
Preterm (<37 weeks)	10 (4.8)	931 (3.1)	64 (2.8)	5 (1.9)	1010 (3.0)		
Sex infant 1, n (%)						0.65402	
Male	105 (50.0)	15568 (51.2)	1177 (50.9)	127 (47.6)	16977 (51.2)		
Female	105 (50.0)	14815 (48.8)	1135 (49.1)	140 (52.4)	16195 (48.8)		
Sex infant 2, n (%)						<.00012	
Male	140 (66.7)	15589 (51.3)	1118 (48.4)	141 (52.8)	16988 (51.2)		
Female	70 (33.3)	14794 (48.7)	1194 (51.6)	126 (47.2)	16184 (48.8)		
Birth weight infant 1, (g)						<.00011	
Mean (SD)	3193.2 (451.19)	3336.8 (507.99)	3450.3 (454.23)	3453.5 (482.09)	3344.8 (504.93)		
Median (IQR)	3180.0 (2940.0, 3465.0)	3340.0 (3050.0, 3640.0)	3450.0 (3150.0, 3750.0)	3475.0 (3120.0, 3825.0)	3350.0 (3050.0, 3644.0)		
Range	1715.0, 4350.0	800.0, 4000.0	1530.0, 4890.0	1645.0, 5050.0	800.0, 4000.0		
Birth weight category infant 1, n (%)						<.00012	
<2kg	2 (1.0)	179 (0.6)	9 (0.4)	1 (0.4)	191 (0.6)		
2kg - 3.99	200 (95.2)	27987 (92.1)	2033 (87.9)	230 (86.1)	30450 (91.8)		
≥4kg	8 (3.8)	2217 (7.3)	270 (11.7)	36 (13.5)	2531 (7.6)		
Birth weight infant 2, (g)						<.00011	
Mean (SD)	3218.7 (474.82)	3466.1 (453.55)	3652.0 (467.69)	3721.2 (484.12)	3479.6 (458.35)		
Median (IQR)	3220.0 (2950.0, 3535.0)	3460.0 (3170.0, 3765.0)	3647.5 (3340.0, 3970.0)	3710.0 (3405.0, 4060.0)	3470.0 (3180.0, 3780.0)		
Range	1465.0, 4630.0	665.0, 5600.0	860.0, 5560.0	2000.0, 5150.0	665.0, 5600.0		
Birth weight category infant 2, n (%)						<.00012	
<2kg	4 (1.9)	83 (0.3)	4 (0.2)	0 (0.0)	91 (0.3)		
2kg - 3.99	197 (93.8)	26776 (88.1)	1771 (76.6)	189 (70.8)	28933 (87.2)		
≥4kg	9 (4.3)	3524 (11.6)	537 (23.2)	78 (29.2)	4148 (12.5)		
Birth weight category infant 1, n (%)						<.00012	
Small for gestational age	33 (15.7)	2428 (8.0)	98 (4.2)	12 (4.5)	2571 (7.8)		
    Appropriate for gestational age	164 (78.1)	24615 (81.0)	1845 (79.8)	208 (77.9)	26832 (80.9)		
Large for gestational age	13 (6.2)	3340 (11.0)	369 (16.0)	47 (17.6%)	3769 (11.4)		
Birth weight category infant 2, n (%)						<.00012	
Small for gestational age	46 (21.9)	2360 (7.8)	67 (2.9)	9 (3.4)	2482 (7.5)		
Appropriate for gestational age	159 (75.7)	24786 (81.6)	1740 (75.3)	173 (64.8)	26858 (81.0)		
Large for gestational age	5 (2.4)	3237 (10.7)	505 (21.8)	85 (31.8)	3832 (11.6)		
Feeding infant 1, n (%)						0.99252	
Exclusively BF at 6 months	15 (7.1)	2007 (6.6)	146 (6.3)	16 (6.0)	2184 (6.6)		
  Exclusively BF at (least) 12 weeks	57 (27.1)	8316 (27.4)	614 (26.6)	74 (27.7)	9061 (27.3)		
Exclusively BF at (least) 6 days	78 (37.1)	11587 (38.2)	886 (38.4)	100 (37.5)	12651 (38.2)		
Not exclusively BF at 6 days	60 (28.6)	8437 (27.8)	663 (28.7)	77 (28.8)	9237 (27.9)		
Feeding infant 2, n (%)						0.36852	
Exclusively BF at 6 months	22 (10.5)	2694 (8.9)	187 (8.1)	18 (6.8)	2921 (8.8)		
Exclusively BF at (least) 12 weeks	54 (25.7)	7558 (24.9)	541 (23.4)	65 (24.4)	8218 (24.8)		
Exclusively BF at (least) 6 days	61 (29.0)	9902 (32.6)	797 (34.5)	96 (36.1)	10856 (32.8)		
Not exclusively BF at 6 days	73 (34.8)	10178 (33.6)	786 (34.0)	87 (32.7)	11124 (33.6)		
BMI child 1 at 2 years (kg/m2)						<.00011	
Mean (SD)	14.9 (1.07)	16.1 (1.29)	17.2 (1.38)	17.6 (1.45)	16.2 (1.33)		
Median (IQR)	14.9 (14.1, 15.5)	16.0 (15.3, 16.9)	17.1 (16.2, 18.0)	17.6 (16.6, 18.6)	16.1 (15.3, 17.0)		
Range	12.4, 18.5	10.9, 54.9	13.4, 26.0	14.4, 21.9	10.9, 54.9		
BMI category child 1 at 2 years, n (%)						<.00012	
Underweight BMI	15 (7.1)	283 (0.9)	1 (0.0)	0 (0.0)	299 (0.9)		
Healthy BMI	193 (91.9)	28447 (93.6)	1834 (79.3)	182 (68.2)	30656 (92.4)		
Overweight BMI	2 (1.0)	1503 (4.9)	401 (17.3)	67 (25.1)	1973 (5.9)		
Obese BMI	0 (0.0)	150 (0.5)	76 (3.3)	18 (6.7)	244 (0.7)		
1Kruskal-Wallis p-value; 2Chi-Square p-value;			

BMI: Body Mass Index, BF: Breastfeeding
